# Association between ultra-processed food consumption and the incidence of type 2 diabetes: the ELSA-Brasil cohort

**DOI:** 10.1186/s13098-023-01162-2

**Published:** 2023-11-15

**Authors:** Scheine L. Canhada, Álvaro Vigo, Renata Levy, Vivian C. Luft, Maria de Jesus M. da Fonseca, Luana Giatti, Maria del Carmen B. Molina, Bruce B. Duncan, Maria Inês Schmidt

**Affiliations:** 1 Postgraduate Program in Epidemiology, Universidade Federal do Rio Grande do Sul, Rua Ramiro Barcelos 2600 sala 519, Porto Alegre, RS Brazil; 2 Department of Preventive Medicine, School of Medicine, Universidade de São Paulo, São Paulo, SP Brazil; 3 Postgraduate Program in Food, Nutrition and Health, UFRGS; Hospital de Clínicas de Porto Alegre, Porto Alegre, RS Brazil; 4 National School of Public Health, Fundação Oswaldo Cruz, Rio de Janeiro, RJ Brazil; 5 Postgraduate Program in Public Health and School of Medicine & Clinical Hospital, Universidade Federal de Minas Gerais, Belo Horizonte, MG Brazil; 6 Postgraduate Program in Nutrition and Health, Universidade Federal do Espírito Santo, Vitória, ES Brazil

**Keywords:** Epidemiology, Non-communicable diseases, Public health, Type 2 diabetes, Ultra-processed food

## Abstract

**Background:**

Ultra-processed food (UPF) consumption increases the risk of type 2 diabetes in various high-income countries, with some variation in the magnitude across studies. Our objective was to investigate the association of UPF consumption and specific subgroups with incident type 2 diabetes in Brazilian adults.

**Methods:**

The Brazilian Longitudinal Study of Adult Health (ELSA-Brasil) is a multicenter cohort study of 15,105 adults (35–74 years) enrolled in public institutions in Brazil (2008–2010). We followed participants with two clinic visits (2012–2014; 2017–2019) and annual telephone surveillance. After excluding those with diabetes at baseline, who died or were lost in the follow-up, with missing data, with implausible energy food intake, or reporting bariatric surgery, there were 10,202 participants. We used the NOVA classification to assess UPF consumption based on a food frequency questionnaire. We defined type 2 diabetes by self-report, medication use, or comprehensive laboratory tests. We estimated relative risks (RR) and 95% confidence intervals (95% CI) using robust Poisson regression.

**Results:**

Median UPF consumption was 372 g/day. Over 8.2 (0.7) years of follow-up, we detected 1799 (17.6%) incident cases. After adjustment for socio-demographics, family history of diabetes, and behavioral risk factors, comparing the fourth (≥ 566 g/day) with the first (< 236 g/day) quartile of UPF distribution, RR was 1.24 (1.10–1.39); every 150 g/day increments in UPF consumption resulted in a RR of 1.05 (1.03–1.07). Reclassifying natural beverages with added sweeteners as UPF increased risk (RR 1.40; 1.25–1.58). Among UPF subgroupings, consumption of processed meats and sweetened beverages increased diabetes risk, while yogurt and dairy sweets decreased the risk (p < 0.05).

**Conclusions:**

UPF consumption increased the incidence of type 2 diabetes in Brazilian adults, with heterogeneity across specific food items. These findings add to previous evidence for the role of UPFs in the development of diabetes and other chronic diseases, supporting recommendations to avoid their intake in diabetes prevention and management.

**Supplementary Information:**

The online version contains supplementary material available at 10.1186/s13098-023-01162-2.

## Background

Ultra-processed foods (UPFs) have received recent attention in nutritional epidemiology. Being nutritionally unbalanced, rich in additives (flavors, emulsifiers, and dyes, among others), and designed to be highly convenient and attractive to stimulate consumption [1], their intake has increased rapidly over the last few decades [2, 3] with crucial adverse health effects. According to a survey conducted in Brazil, the consumption of UPF accounted for approximately 20% of the total diet, with bread, non-dairy sweets and desserts, processed meats, and sodas being the main contributors [4].

Greater consumption of UPFs relates to higher overall mortality and the incidence of various non-communicable chronic diseases, including cancer and cardiovascular diseases [5, 6]. It also predicted weight [5, 7, 8] and waist [8] gains. In addition, six prospective studies in European and North American countries [9–13] have found that higher consumption of UPFs predicted an increased risk of type 2 diabetes. Based on cohort studies in the United States, one report [13] identified animal-based products, ready-to-eat/heat-mixed dishes, and artificially and sugar-sweetened beverages as the UPF subgroups most related to developing type 2 diabetes.

Eating patterns vary across populations, and the interactions of UPFs with traditional eating patterns in different cultures and their insertion in different nutritional transitions may produce distinct health effects. Thus, it is noteworthy that this association has not been evaluated in low and middle-income countries, where traditional dietary patterns may differ, consumption of UPFs is lower, and where 95% of new cases of diabetes will occur by 2030 [14]. Thus, we aimed to investigate the association between UPF food consumption and the risk of developing type 2 diabetes in Brazil, a large middle-income country currently ranked 6th in the number of persons living with diabetes globally. To this end, we analyzed a cohort of Brazilian adults, the ELSA-Brasil study.

## Methods

### Study design and population

The Brazilian Longitudinal Study of Adult Health (in Portuguese, ‘*Estudo Longitudinal de Saúde do Adulto*,’ ELSA-Brasil) is a multicenter prospective occupational cohort aiming primarily to address risk factors for the development and progression of chronic diseases, particularly cardiovascular diseases and diabetes, over a long-term follow-up [15]. Ethics committees of each institution approved the research protocol, and subjects gave written consent to participate in each visit.

Between August 2008 and December 2010, we recruited 15105 active or retired, non-pregnant civil servants, aged 35–74 years, from public institutions of higher education and research located in six Brazilian capital cities (Salvador, Belo Horizonte, Rio de Janeiro, São Paulo, Vitoria, and Porto Alegre), and applied a series of questionnaires as well as laboratory and clinical examinations [15–17]. Participants returned twice to the study sites (2012–2014 and 2017–2019) for further investigation. Additionally, they have responded to annual telephone surveillance since 2009.

Among the 15105 participants enrolled, we excluded those with prevalent diabetes at baseline (n = 2429), with implausible food intake (< 600 kcal/d or > 6000 kcal/d) (n = 197), who died (n = 321), were lost to follow-up (n = 1361), had missing data on variables of interest (n = 484), or had bariatric surgery between visits (n = 111). The final sample had 10202 participants (Additional file 1: Fig. S1).

### Baseline measurements

All measurements followed standardized protocols and regular quality control assessments [18]. In each visit, after an overnight fast, we measured weight, height, and waist circumference following internationally standardized protocols and defined body mass index (BMI) as weight (kg)/height (m)^2^. We also obtained a fasting blood sample by venipuncture and conducted a standardized 2-h 75-g oral glucose tolerance test (WHO 1999). Plasma glucose was measured using hexokinase and HbA1c by high-pressure liquid chromatography (Bio-Rad, certified by the National Glycohemoglobin Standardization Program).

We interviewed participants using structured questionnaires to ascertain age, sex, self-declared race/color, educational achievement, family income, previous medical history, smoking (current and previous), alcohol consumption, physical activity, and family history of diabetes.

Food consumption was evaluated at baseline through a previously validated food frequency questionnaire, with 114 food items [19]. For each item, we obtained the frequency of consumption in the last year (with eight response options: ‘more than 3 times/day’, ‘2–3 times/day’, ‘once daily’, ‘5–6 times/week’, ‘2–4 times/week’, ‘once/week’, ‘1–3 times/month’ and ‘never/almost never’) and the number of portions consumed, using standardized portion sizes. We then calculated the daily amount consumed for each food item in grams by multiplying its portion number, weight, and frequency. Next, we estimated the nutritional composition and energy using the University of Minnesota Nutrition Data System for Research (NDSR) software. Finally, we imputed the 99^th^ percentile value for a food item when that food's value was above the 99^th^ percentile of its distribution.

### Definition of ultra-processed foods

**Fig. 1:**
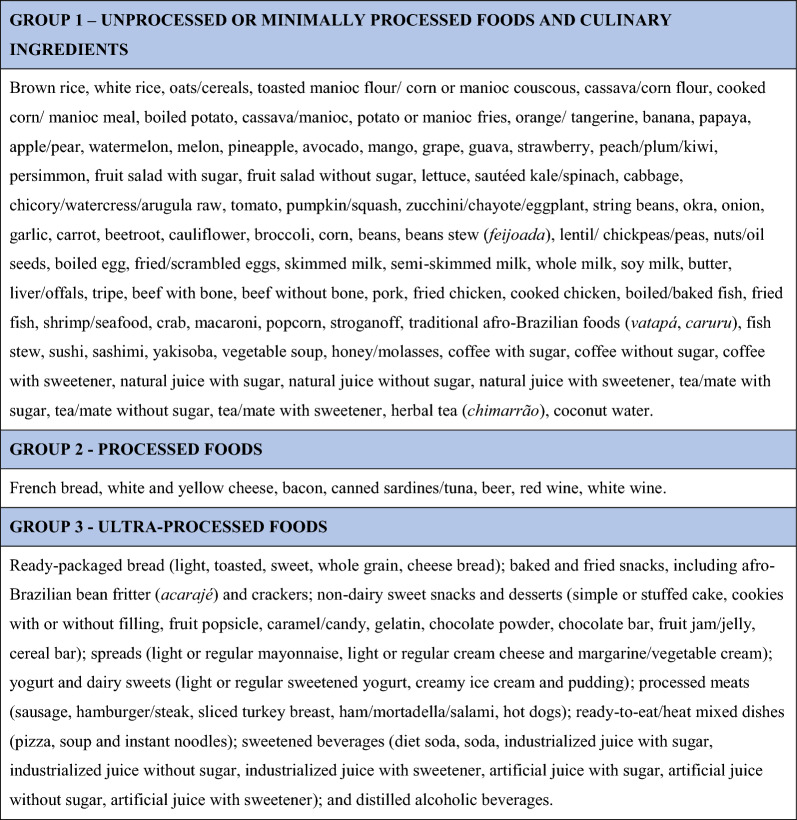
Box of ELSA-Brasil food subgroups according to the NOVA classification, based on the degree of food industrial processing

We grouped food items according to the extent and purpose of their industrial processing (the NOVA classification): (i) non- or minimally processed foods and culinary ingredients, (ii) processed foods, and (iii) ultra-processed foods [1]. We aggregated non- or minimally processed foods and culinary ingredients into a single group because our food frequency questionnaire did not distinguish culinary ingredients from the food items that included them (Fig. 1).

In secondary analyses we classified artificially sweetened natural juices, coffee, or tea as UPFs (rather than non- or minimally-processed foods and culinary ingredients), given our previous findings suggesting increased diabetes risk in certain groups [20].

### Outcomes

We ascertained diabetes based on laboratory measurements taken during visits and reports from the annual telephone follow-up surveillance. We defined as diabetes cases those who: (i) reported a medical diagnosis of diabetes or current use of medication for diabetes, or (ii) had a laboratory measurement reaching the thresholds for fasting plasma glucose (FPG) (≥ 7.0 mmol/L; 126 mg/dL), 2 h post-load glucose (PG) (≥ 11.1 mmol/L; 200 mg/dL), or HbA1c (≥ 48 mmol/mol; 6.5%) (WHO 2006; ADA 2014). We excluded prevalent diabetes at baseline and ascertained incident diabetes at follow-up visits based on these criteria. We additionally included new cases reporting a diagnosis of diabetes on at least two annual telephone interviews after the last clinic visit.

### Statistical analysis

We describe participant characteristics and outcomes using absolute and relative frequencies for categorical variables and mean, standard deviation or median and 25th–75th percentiles for continuous variables. To assess the statistical significance of differences between means or proportions, we employed ANOVA and Chi-square tests, respectively.

We characterized UPF consumption in two ways: first, using mean consumption in grams per day (g/day), expressed as a mean difference of 150 g/day, which represents approximately a 10% difference in consumption in our sample; and second, creating quartiles of consumption (g/day). We used grams instead of kcal to express quantity because some foods and beverages in the UPF group do not provide energy.

We examined the shape of the association along the continuum of UPFs using restricted cubic splines and tested the non-linearity of the associations [21]. We estimated relative risks (RR) and 95% confidence intervals (95% CI) using robust Poisson regression to investigate the associations of UPF intake with incident diabetes. Progressively adjusted models included: in model 2, age (in years), sex (male or female), race/color (white, brown, black, Asian, or Indigenous), school achievement (less than elementary, elementary, secondary or college/university), per capita family income (in Brazilian currency, *reais*), family history of diabetes (yes or no), smoking (never, former or current), physical activity (in MET minutes/week), and alcohol consumption (in grams/week); in model 3a, model 2 plus energy intake (in kilocalories/day); in model 3b, model 2 plus hypertension (yes or no); and in model 3c, model 2 plus BMI (in kg/m^2^). We considered model 2 as our final adjustment model; models 3a-c permit a comparison of our results with those of other studies. We drew directed acyclic graphs to understand the variable relationships (Additional file 1: Figure S2).

We tested potential multiplicative interactions by adding multiplicative terms to models. The interactions considered were intermediate hyperglycemia (yes or no), self-reported recent diet change (yes or no), sex (male or female), fruit and vegetable consumption (in grams/day), and (model 3c) with BMI (kg/m^2^). We assessed multicollinearity between variables, setting a limit of 2 for the variance inflation factor. The overall fit of the model to the data was assessed using the method described by Hosmer and Lemeshow [22].

To assess the robustness of our findings, we performed several sensitivity analyses, based on model 2, by (i) reclassifying natural juices and coffee/tea with added artificial sweeteners from the group of non- or minimally processed foods and culinary ingredients to the UPF group, (ii) alternatively quantifying UPFs as a proportion of grams relative to the total daily grams consumed, (iii) including red meat intake, (iv) fruits and vegetable intake, (v) saturated fat intake, (vi) sugar intake, (vii) fiber intake, and (viii) diet quality as adjustment variables [23], (ix) including 16 cases of incident diabetes who died and were thus excluded in the original analyses, and, finally, (x) performing multiple imputation to permit the addition in analyses of participants with missing values in covariates.

We also evaluated the association between the consumption of specific UPF subgroups and the incidence of diabetes using model 2 for increments of 50 g/d and one standard deviation in each group. We conducted all analyses with the statistical software package SAS Studio® (SAS OnDemand for Academics).

## Results

Table 1 provides a detailed description of the sample, overall and according to quartiles of UPF consumption. Briefly, 5846 (57.3%) were women, 5597 (54.9%) were self-declared as being white, 5871 (57.6%) had a complete college/university degree, and 3675 (36%) had a family history of diabetes. Median and IQR values were 372 (235–565) g/day for UPF consumption, 50 (44–57) years for age, and 25.9 (23.4–28.9) kg/m^2^ for BMI. Compared with those in the first UPF quartile (< 236 g/day), participants in the fourth quartile (≥ 566 g/day) were younger and had higher consumption of overall energy, red meat, saturated fat, and sugar, as well as higher BMI. Those in the top quartile were less frequently women and more frequently white.

**Table 1:** Characteristics of the study sample according to ultra-processed food (UPF) consumption. ELSA-Brasil, 2008–2010 (N = 10202)

	Quartile 1^1^ (n = 2550)		Quartile 2 (n = 2551)		Quartile 3 (n = 2551)		Quartile 4 (n = 2550)			Total (n = 10202)	
	Median or N^2^	IQR or %	Median or N	IQR or %	Median or N	IQR or %	Median or N	IQR or %	P value	Median or N	IQR or %
Ultra-processed foods (g/d)	166	120–204	301	267–335	453	412–504	758	652–940	< 0.0001	372	235–565
Age (years)	53	47–59	50	44–57	49	44–55	48	43–54	< 0.0001	50	44–57
Sex									< 0.0001		
Female	1516	59.5	1564	61.3	1501	58.8	1265	49.6		5846	57.3
School achievement									< 0.0001		
Less than elementary	140	5.5	74	2.9	64	2.5	92	3.6		370	3.6
Elementary	158	6.2	116	4.5	121	4.7	146	5.7		541	5.3
Secondary	832	32.6	823	32.3	864	33.9	901	35.3		3420	33.5
College/University	1420	55.7	1538	60.3	1502	58.9	1411	55.3		5871	57.6
Income (*reais*)	1380	726–2282	1522	913–2352	1452	747–2352	1452	726–2075	0.09	1452	726–2282
Race/color									< 0.0001		
Black	429	16.8	322	12.6	349	13.7	376	14.7		1476	14.5
Brown	745	29.2	737	28.9	686	26.9	622	24.4		2790	27.3
White	1256	49.3	1419	55.6	1446	56.7	1476	57.9		5597	54.9
Asian	91	3.6	53	2.1	54	2.1	49	1.9		247	2.4
Indigenous	29	1.1	20	0.8	16	0.6	27	1.1		92	0.9
Family history of diabetes	934	36.6	912	35.8	892	35.0	937	36.8	0.51	3675	36.0
Smoking									0.05		
Never	1520	59.6	1591	62.4	1534	60.1	1505	59.0		6150	60.3
Former	733	28.8	687	26.9	708	27.8	703	27.6		2831	27.7
Current	297	11.7	273	10.7	309	12.1	342	13.4		1221	12.0
Physical activity (METs/w)	297	0–960	330	0–1056	240	0–960	198	0–891	0.04	264	0–960
Alcohol (g/w)	9	0–69.9	0	0–64.0	0	0–62.6	0	0–65.6	0.01	0	0–65.6
Hypertension	843	33.1	706	27.7	660	25.9	730	28.6	< 0.0001	2939	28.8
Energy intake (kcal/d)	1985	1610–2461	2280	1882–2800	2596	2132–3158	3103	2459–3878	<0 .0001	2450	1946–3120
Red meat (g/d)	42	19–80	42	30–80	46	40–89	65	40–102	< 0.0001	45	36–88
Fruits and vegetables (g/d)	434	276–621	427	280–625	440	291–637	432	278–650	0.004	432	281–632
Saturated fat (g/d)	21	16–27	26	20–33	30	24–39	37	28–48	< 0.0001	28	21–37
Fiber (g/d)	25	19–33	27	21–36	29	22–38	32	24–43	< 0.0001	28	21–38
Sugar (g/d)	84	63–112	102	80–131	121	95–156	158	118–203	< 0.0001	113	83–154
BMI (kg/m^2^)	25.5	23.1–28.2	25.4	23.1–28.6	26.0	23.4–28.8	26.7	24.0–29.9	< 0.0001	25.9	23.4–28.9
T2DM incidence^3^	459	18.0	404	15.8	425	16.7	511	20.0	0.0005	1799	17.6

^1^UPF consumption: quartile 1 ≤ 235 g/day, quartile 2 236–372 g/day, quartile 3 373–565 g/day, and quartile 4 ≥ 566 g/day

^2^Quantitative variables are expressed as the median and interquartile range (IQR), and categorical variables as the number of people (N) and percentage (%)

^3^T2DM = Type 2 diabetes mellitus

**Fig. 2:**
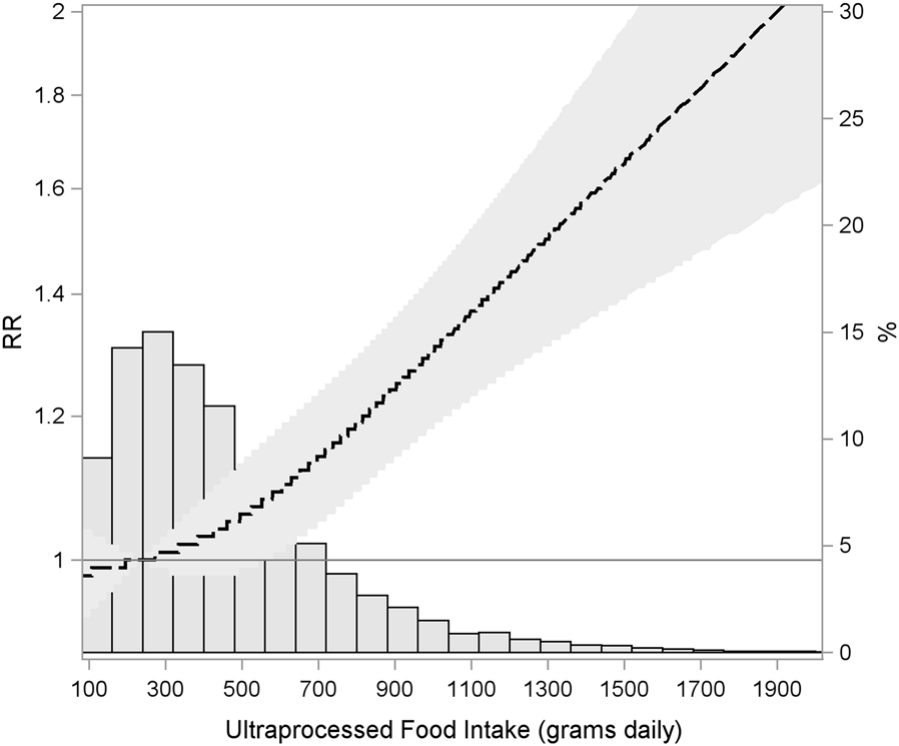
Restricted cubic splines for the association between ultra-processed food consumption and incidence of type 2 diabetes, adjusted for model 2. The dashed line shows the point estimates of relative risk (RR) for the association along the spectrum of ultra-processed food consumption and the stippled area, the 95% confidence limits. The histogram shows the distribution of ultra-processed foods consumption (% of the study sample; right vertical axis)

After 8.2 (± 0.7) years of follow-up, 1799 (17.6%, 95%CI 16.9–18.4%) participants developed type 2 diabetes. Restricted cubic spline regression models showed a linear relative increase in the incidence of type 2 diabetes with rising values of UPF consumption when adjusting for age, sex, race/color, income, school achievement, family history of diabetes, smoking, physical activity, and alcohol (p non-linearity = 0.20) (Fig. 2).

Table 2 shows the results of the association between UPF consumption and the incidence of type 2 diabetes. When comparing the fourth (≥ 566 g/day) with the first quartile (< 236 g/day) of UPF consumption and adjusting for sociodemographic, clinical, and behavioral risk factors, we found a RR of 1.24 (95% CI 1.10–1.39, model 2, main analysis). For every 150 g/day increment in UPF consumption, we found a RR of 1.05 (95% CI 1.03–1.07, model 2). Results after adding the potential mediators of energy intake and hypertension (models 3a, b) changed the magnitude of the associations only slightly while adding BMI (model 3c) led to a marked reduction.

**Table 2:** Association of ultra-processed food consumption with the incidence of diabetes, ELSA-Brasil (N = 10,202)

	Ultra-processed food consumption								
	Quartile 1^1^ ≤ 235 g/day	Quartile 2 236–372 g/day		Quartile 3 373–565 g/day		Quartile 4 ≥ 566 g/day		For a 150 g/day increase	
Models^2^	RR	RR	95% CI	RR	95% CI	RR	95% CI	RR	95% CI
**1**	1	0.88	0.78, 0.99	0.93	0.82, 1.04	1.11	0.99, 1.25	1.04	1.02, 1.06
**2**	1	0.96	0.85, 1.08	1.03	0.92, 1.16	1.24	1.10, 1.39	1.05	1.03, 1.07
**3a**	1	0.97	0.86, 1.10	1.06	0.93, 1.20	1.29	1.13, 1.47	1.07	1.04, 1.09
**3b**	1	0.97	0.86, 1.09	1.04	0.92, 1.17	1.22	1.09, 1.37	1.05	1.03, 1.07
**3c**	1	0.93	0.83, 1.05	0.96	0.85, 1.08	1.09	0.97, 1.22	1.02	1.00, 1.05

^1^Quartile 1: Reference

^2^Model 1: Non-adjusted

Model 2: Age, sex, race/color, income, school achievement, family history of diabetes, smoking, physical activity and alcohol

Model 3a: 2 + Energy intake

Model 3b: 2 + Hypertension

Model 3c: 2 + BMI

We found no multicollinearity among the variables included nor evidence to reject the global fit of the model to the data (p = 0.31). We did not observe effect modification by BMI, intermediate hyperglycemia, self-reported recent change in diet, sex, and fruit and vegetable consumption (p-values of 0.32, 0.95, 0.74, 0.20, and 0.91, respectively).

Table 3 shows the association of UPF subgroups consumption, expressed continuously for 50 g/day and a one SD difference, with the incidence of diabetes. With the division of UPF into smaller groups, only a few associations presented statistical significance. Processed meats and sweetened beverages increased the adjusted risk of diabetes in both analyses. For processed meat, the relative risk for a one SD (21 g) difference was 1.08 (95% CI 1.04–1.13). For sweetened beverages, the relative risk for a one SD (230 ml) difference was 1.14 (95% CI 1.10–1.18). Greater consumption of yogurt and dairy sweets decreased risk in both analyses, with a 61 g/day difference having an adjusted relative risk of 0.94 (95% CI 0.89–0.98).

**Table 3:** Association of ultra-processed food (UPF) subgroups consumption with the incidence of diabetes, ELSA-Brasil (N = 10,202)

	For a 50 g/day increase					For a SD/day increase			
	Crude		Adjusted^1^		SD	Crude		Adjusted^1^	
UPF Subgroups	RR	95% CI	RR	95% CI		RR	95% CI	RR	95% CI
1. Ready-packaged bread	0.95	0.91, 1.00	0.96	0.92, 1.01	48 g	0.95	0.91, 1.00	0.97	0.92, 1.01
2. Baked and fried snacks	0.94	0.85, 1.04	0.93	0.84, 1.04	21 g	0.97	0.93, 1.02	0.97	0.93, 1.02
3. Non-dairy sweet snacks and desserts	0.99	0.95, 1.03	0.98	0.94, 1.03	56 g	0.99	0.94, 1.04	0.98	0.94, 1.03
4. Spreads	0.95	0.78, 1.16	0.99	0.82, 1.19	12 g	0.99	0.94, 1.04	1.00	0.95, 1.04
5. Yogurt and dairy sweets	0.93	0.89, 0.96	0.95	0.91, 0.98	61 g	0.91	0.87, 0.95	0.94	0.89, 0.98
6. Processed meats	1.13	1.02, 1.25	1.20	1.09, 1.32	21 g	1.05	1.01, 1.10	1.08	1.04, 1.13
7. Ready-to-eat/heat mixed dishes	0.93	0.84, 1.03	0.99	0.90, 1.08	25 g	0.96	0.92, 1.01	0.99	0.95, 1.04
8. Sweetened beverages	1.03	1.02, 1.04	1.03	1.02, 1.04	230 ml	1.14	1.10, 1.18	1.14	1.10, 1.18
9. Distilled alcoholic beverages	1.31	1.05, 1.63	1.14	0.89, 1.48	8 g	1.04	1.01, 1.08	1.02	0.98, 1.06

^1^Adjusted according to Model 2 of Table 2: Age, sex, center, race/color, income, school achievement, family history of diabetes, smoking, physical activity and alcohol

SD = standard deviation

We also conducted sensitivity analyses (Table 4). When classifying artificially sweetened natural juices, coffee, or tea as UPFs (rather than non- or minimally-processed foods and culinary ingredients), associations increased (third quartile RR = 1.32, 95% CI 1.17–1.49, fourth quartile RR = 1.40, 95% CI 1.25–1.58). All other analyses produced estimates similar to those presented in Table 2.

**Table 4:** Sensitivity analyses for the association of ultra-processed (UPF) consumption with incident diabetes (n = 10,202)

	Absolute increments	Quartile 2	Quartile 3	Quartile 4
	RR (95% CI)	RR (95% CI)	RR (95% CI)	RR (95% CI)
Reclassifying natural juice/coffee/tea with added sweeteners as UPFs rather than as non- or minimally processed food	1.06 (1.04, 1.08)	1.04 (0.92, 1.18)	1.32 (1.17, 1.49)	1.40 (1.25, 1.58)
Defining daily UPF consumption as a proportion of the daily diet´s weight	1.06 (1.03, 1.10)	1.04 (0.92, 1.17)	1.09 (0.96, 1.23)	1.24 (1.10, 1.40)
Inclusion of red meat intake as an adjustment variable	1.05 (1.03, 1.07)	0.96 (0.85, 1.08)	1.02 (0.91, 1.15)	1.20 (1.07, 1.35)
Inclusion of fruit and vegetable intake as an adjustment variable	1.05 (1.03, 1.07)	0.96 (0.85, 1.08)	1.03 (0.91, 1.16)	1.23 (1.09, 1.38)
Inclusion of saturated fat intake as an adjustment variable	1.07 (1.05, 1.09)	0.98 (0.87, 1.11)	1.07 (0.95, 1.21)	1.32 (1.16, 1.50)
Inclusion of sugar intake as an adjustment variable	1.09 (1.06, 1.11)	0.99 (0.87, 1.11)	1.09 (0.96, 1.23)	1.37 (1.20, 1.56)
Inclusion of fiber intake as an adjustment variable	1.06 (1.04, 1.08)	0.97 (0.86, 1.09)	1.05 (0.93, 1.18)	1.27 (1.13, 1.43)
Inclusion of diet quality as an adjustment variable	1.06 (1.04, 1.09)	0.97 (0.86, 1.10)	1.05 (0.93, 1.19)	1.29 (1.15, 1.46)
Inclusion of 16 cases of incident diabetes who died and were excluded among deaths	1.05 (1.03, 1.07)	0.96 (0.85, 1.09)	1.03 (0.92, 1.17)	1.24 (1.11, 1.39)
Multiple imputation	1.04 (1.02, 1.06)	0.96 (0.86, 1.08)	1.02 (0.91, 1.15)	1.20 (1.07, 1.34)

All analyses were performed with Model 2, as defined in Table 2

Absolute increments: 7% was used for the “UPF consumption as a proportion of the daily diet´s weight” (10th percentile of consumption). 150 g/d increases were used for all the other analyses

New quartiles for UPF consumption as a proportion of the daily diet´s weight analysis: quartile 1: reference; quartile 2: 10.8–16.2%; quartile 3: 16.3–23.4%; quartile 4: ≥ 23.5%

New quartiles for reclassification analysis: quartile 1: reference; quartile 2: 273–436 g/day; quartile 3: 437–669 g/day; quartile 4: ≥ 670 g/day

## Discussion

Greater consumption of UPFs increased the incidence of type 2 diabetes over an average of eight years of follow-up in our sample of middle-aged and elderly Brazilian adults. The association was linear over a relevant consumption range and was evident both when comparing extreme quartiles of UPF consumption and increases of 150 g/day in consumption. Within the UPF food subgroups, sweetened beverages and processed meat presented the strongest associations; however, consuming yogurt and dairy sweets was protective.

Our findings align with those of previous observational studies relating UPFs to the incidence of diabetes in France, United Kingdom, Spain, Netherlands, and United States [9–13]. When comparing the extremes of UPF consumption, the relative risks (RRs) varied across studies, from 1.19 (as observed in the Nurses’ Health Study conducted in the US) to 1.56 (in the Lifelines cohort from the Netherlands). Differences may result from specific modeling strategies and population characteristics, and from varying follow-up times, as outlined in Additional file 1: Table S1.

We found weaker associations, not statistically significant after the inclusion of BMI, a potential mediator. We cannot fully explain our smaller associations, but some aspects merit consideration. First, our definition of incident diabetes included not only self-reported information but also laboratory determinations made at a single moment, thus being more sensitive and less specific than the definitions in many other studies. This definition may have diluted the magnitude of our associations. Second, our food frequency questionnaire, not designed to assess UPFs, may have resulted in greater imprecision in our characterization of UPF consumption, biasing the association toward the null. Third, the somewhat stronger association found after reclassification of natural juice/coffee/tea with added sweeteners as UPFs and not as unprocessed or minimally processed foods (sensitivity analyses) suggests that our primary analyses may underestimate the UPF/diabetes association in Brazilian adults because using sweeteners in coffee and natural juices is widespread in Brazil. Finally, other specific aspects of the Brazilian diet may explain the smaller association, although we have adjusted for multiple dietary factors, including an indicator of a healthy diet.

We considered some possibilities to explain the role of UPFs in type 2 diabetes development. UPFs are rich in calories, which can lead to weight gain and obesity, and have a poor nutritional value—high contents of saturated fat, sugar, and sodium, high glycemic index [24], and low content of fiber [25]. However, adjustment for most of these dietary factors, including diet quality, led to an increase in the associations, not a decrease. Although including BMI in the model decreased associations considerably, we cannot distinguish whether this resulted from confounding or from mediation, as adiposity impacts diabetes as a continuous and chronic process.

Thus, additional potential mechanisms of UPFs deserve attention. UPFs contain substances not used in traditional food preparation (such as trans-fat) and numerous additives (such as emulsifiers, nonnutritive sweeteners, and thickeners), some of which have been associated with cardiometabolic effects [26, 27]. Emulsifiers, a common additive in UFPs, have been shown to disrupt the intestinal mucus barrier, leading to the greater passage of pro-inflammatory stimulants, which may further stimulate the development of diabetes [28]. Concerning nonnutritive sweeteners, a recent randomized clinical trial showed them to produce impaired glycemic responses by apparently acting through alteration in the intestinal microbiota [29]. UPFs also increase the intake of substances such as bisphenol A, which migrates into foods from UPF packaging and has been associated with type 2 diabetes incidence [30, 31].

Our findings regarding specific components of UPFs relating differently to the development of type 2 diabetes generally align with those found in cohorts of health professionals in the United States [13]. Processed meats and sweetened beverages were the main drivers of the increased risk of type 2 diabetes in our sample. Meta-analyses of cohort studies support our results [32, 33]. On the contrary, yogurt and dairy sweets acted as protective factors despite being mostly industrialized and with added sugar or artificial sweeteners. Diabetes protection from dairy products is not surprising since meta-analyses in different populations [34, 35] and a previous study from ELSA-Brasil [36] also indicate protection. Unfortunately, these studies do not distinguish dairy products with and without added sugar, and further studies are necessary to unravel these issues.

Though some UPF food subgroups showed positive associations with incident diabetes while others did not, we continue to support the concept that UPFs as a dietary pattern characterized by the NOVA classification are likely harmful. In addition to increasing all-cause mortality, UPFs have been shown to increase the risk of various diseases, ranging from obesity and other cardiometabolic ones to cancer, irritable bowel disease, frailty, and depression [37]. In addition, the UPF concept facilitates public health messaging on nutrition. Further studies of the association of UPF subgroups, particularly yogurt and dairy desserts, with diabetes and the other chronic conditions associated with greater UPF consumption are necessary.

Our study has limitations. First, our food frequency questionnaire was not specifically designed for the NOVA classification. Although these questionnaires are commonly used to assess nutritional intake in epidemiological studies, our lack of specificity in identifying UPFs may lead to an underestimation of the size of the associations reported. Furthermore, it reflects only partially the large amount of UPFs available today. However, the quantity of ultra-processed foods consumed in this cohort is in line with that of a nationwide representative survey assessed with detailed food registries [4]. Second, our approximately eight-year follow-up may be too short to evaluate the total contribution of UPFs to the development of a chronic condition such as diabetes. Third, although we performed multiple adjustments for possible confounders in statistical analyses, we cannot rule out residual or unmeasured confounding, particularly since some potential mediators may also be potential confounders. Fourth, since our cohort started at age 35, we cannot extrapolate our findings to younger groups. Finally, although, following the design of most cohort studies, we did not randomly draw our sample from the Brazilian adult population, it captures Brazil’s racial, social, and regional diversity [17].

Our study also has strengths. To our knowledge, this is the first report of an association between UPF intake and incident type 2 diabetes originating from a country whose food culture is quite different from those of the northern hemisphere high-income countries from which the association has been previously reported. Thus, our findings support the robustness of the association across varying dietary patterns resulting from introducing UPFs within the context of diverse culinary traditions. Our multiple sensitivity analyses also attest to the robustness of the associations found. Finally, our cohort´s follow-up was excellent, and the comparison of results with and without multiple imputation of missing values in covariates attest to the small possibility of bias.

In conclusion, we provide further prospective evidence that consumption of ultra-processed foods and beverages, particularly processed meat and sweetened beverages and foods, is related to the development of type 2 diabetes in adults. Furthermore, our findings from this Brazilian population extend the UPF risk documentation in northern hemisphere high-income countries to different settings and population dietary patterns.

### Supplementary Information

**Additional file 1: Figure S1.** Flowchart of the analytical sample. **Figure S2.** Directed acyclic graph for the association between ultra-processed food consumption and the incidence of type 2 diabetes. **Table S1. **Studies reporting the association between ultra-processed food (UPF) consumption and the incidence of type 2 diabetes.

## Data Availability

The datasets supporting the conclusions of this article will be made available upon request pending. Considering guidelines placed by the ethics committees responsible for ELSA's study centers, the data used in this study can be made available by a request to ELSA's Publications Committee (pal@ups.br). Additional information can be obtained from the ELSA Coordinator from the Research Center of Rio Grande do Sul (maria.schmidt@ufrgs.br).

## Abbreviations

PG: 2H post-load glucose
BMI: Body mass index
CI: Confidence interval
ELSA-Brasil: *Estudo Longitudinal de Saúde do Adulto*
FPG: Fasting plasma glucose
g: Grams
IQR: Interquartile range
NDSR: Nutrition Data System for Research
RR: Relative risk
T2DM: Type 2 diabetes
UPF: Ultra-processed food
