# Supplementary material for: Association between ultra-processed food consumption and the incidence of type 2 diabetes: the ELSA-Brasil cohort
Source: Diabetol Metab Syndr. 2023 Nov 15;15:233. doi: 10.1186/s13098-023-01162-2 (PMC10647077; doi:10.1186/s13098-023-01162-2)
Supplement: Supplementary file 1 — Additional file 1: Figure S1. Flowchart of the analytical sample. Figure S2. Directed acyclic graph for the association between ultra-processed food consumption and the incidence of type 2 diabetes. Table S1. Studies reporting the association between ultra-processed food (UPF) consumption and the incidence of type 2 diabetes. [file 13098_2023_1162_MOESM1_ESM.docx]

**Association between Ultra-Processed Food Consumption and the Incidence of Type 2 Diabetes – the ELSA-Brasil Cohort**

**Additional File**


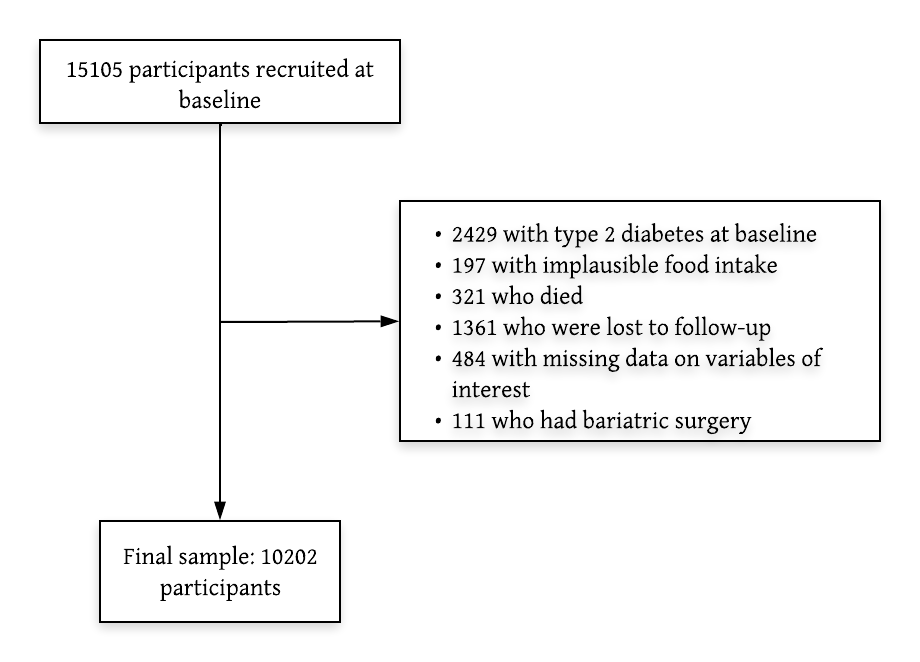


**Figure S1.** Flowchart of the analytical sample.

**
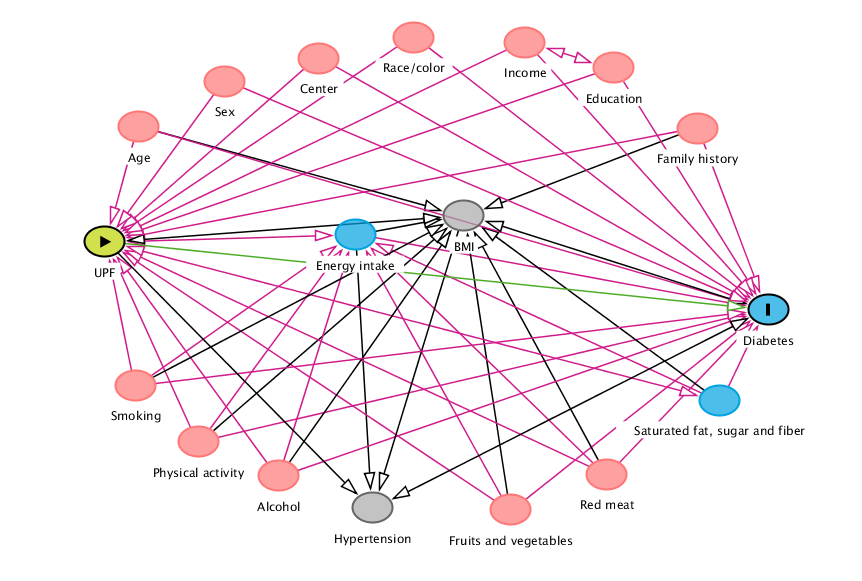
**

**Figure S2.** Directed acyclic graph for the association between ultra-processed food consumption and the incidence of type 2 diabetes.

| **Table S1.** Studies reporting the association between ultra-processed food (UPF) consumption and the incidence of type 2 diabetes. | | | | | | | |
| --- | --- | --- | --- | --- | --- | --- | --- |
| **Study, year of publication** | **Age, range and/or central estimate** | **UPF assessment and consumption** | **Method for T2DM definition** | **Duration of follow-up** | **Models for covariate adjustments** | **Absolute increment in UPF intake** | **Comparing UPF consumption extremes** |
|  |  |  |  |  |  |  |  |
| **NutriNet-Santé, 2020** | ≥18 years  42.7 years | 24-h dietary record  17.3% | Self-reported and confirmed by medical-administrative databases | 6 years | Age, sex, educational level, BMI, physical activity level, smoking status, alcohol intake, number of 24-hour dietary records, energy intake, family history of T2DM, and percentage of weight change. | 1.13 (1.01-1.27) |  |
|  |  |  |  |  |  |  |  |
| **UK Biobank, 2021** | 40-69 years  55.8 years | 24-h dietary recall  22.1% | Self-reported | 5.4 years | Stratification by sex and ethnicity; family history of T2DM, Index of Multiple Deprivation, physical activity level, current smoking status, total energy intake, and BMI. | 1.12 (1.04-1.20) | 1.44 (1.04-2.02) |
|  |  |  |  |  |  |  |  |
| **SUN, 2021** | Range not informed  37.4 years | Food frequency questionnaire 9.5% | Self-reported and confirmed by medical records | 12 years | Sex, age, BMI, educational status, family history of diabetes, smoking status, snacking between meals, active + sedentary lifestyle score, and following a special diet at baseline. Stratified by decades of age and recruitment period. |  | 1.53 (1.06-2.22) |
|  |  |  |  |  |  |  |  |
| **Lifelines, 2022** | 35-70 years  49.1 years | Food frequency questionnaire  34.9% | Self-reported or laboratory measurements | 3.4 years | Age, sex, diet score, total energy intake, alcohol intake, smoking status, educational level, physical activity level, TV watching time, and BMI. | 1.17 (1.09-1.26) | 1.56 (1.27-1.92) |
|  |  |  |  |  |  |  |  |
| **Nurses’ Health Study, 2023** | 30-55 years  Central estimate not informed | Food frequency questionnaire 36.1% | Self-reported and confirmed by a validated questionnaire | 32 years | Age, race/ethnicity, family history of diabetes, history of hypercholesterolemia, history of hypertension, smoking status, physical activity, postmenopausal hormone use, physical examination, neighborhood income, total energy, total alcohol consumption, and BMI. | 1.02 (1.01-1.03) | 1.19 (1.09-1.30) |
|  |  |  |  |  |  |  |  |
| **Nurses’ Health Study II, 2023** | 25-42 years Central estimate not informed | Food frequency questionnaire 36.1% | Self-reported and confirmed by a validated questionnaire | 26 | Age, race/ethnicity, family history of diabetes, history of hypercholesterolemia, history of hypertension, smoking status, physical activity, oral contraceptive use, postmenopausal hormone use, physical examination, neighborhood income, total energy, total alcohol consumption, and BMI. | 1.04 (1.03-1.05) | 1.46 (1.33-1.60) |
|  |  |  |  |  |  |  |  |
| **Health Professional Follow-up Study, 2023** | 40-75 years Central estimate not informed | Food frequency questionnaire 36.1% | Self-reported and confirmed by a validated questionnaire | 30 | Age, race/ethnicity, family history of diabetes, history of hypercholesterolemia, history of hypertension, smoking status, physical activity, physical examination, neighborhood income, total energy, total alcohol consumption, and BMI. | 1.02 (1.01-1.04) | 1.22 (1.07-1.39) |
|  |  |  |  |  |  |  |  |
|  |  |  |  |  |  |  |  |
| **ELSA-Brasil, 2023** | 35-74 years  50.7 years | Food frequency questionnaire 18% | Self-reported and/or laboratory measurements | 8.2 years | Age, sex, race/color, income, school achievement, family history of diabetes, smoking, physical activity, and alcohol. | 1.05 (1.03- 1.07) | 1.24 (1.10-1.39) |
|  |  |  |  |  |  |  |  |
|  |  |  |  |  | Plus energy intake. | 1.07 (1.04- 1.09) | 1.29 (1.13-1.47) |
|  |  |  |  |  |  |  |  |
|  |  |  |  |  | Plus hypertension. | 1.05 (1.03- 1.07) | 1.22 (1.09-1.37) |
|  |  |  |  |  |  |  |  |
|  |  |  |  |  | Plus BMI. | 1.02 (1.00- 1.05) | 1.09 (0.97-1.22) |
|  |  |  |  |  |  |  |  |

UPF consumption is expressed as % of total grams/day. For Nurses’ Health Study, Nurses’ Health Study II, and the Health Professional Follow-up Study: in the original article, the data is presented for all three cohorts together.

The column of ‘Absolute increment in UPF intake’ is expressed as in 10% increments of grams per day, except for the Nurses’ Health Study, Nurses’ Health Study II, and the Health Professional Follow-up Study, which are presented as for 1 serving/day increment.

The comparison between extremes of UPF consumption (quintiles, quartiles, or terciles) was as follows: for UK Biobank, 41.9% vs 7.7% of total grams per day; for SUN project, >323.3 vs <214.6 g/day; for Lifelines, 48.7% vs 23.7% of total g/day; for Nurses’ Health Study, 10.5 vs 3.3 servings/day; for Nurses’ Health Study II, 11.2 vs 3.6 servings/day; for Health Professional Follow-up Study, 11.2 vs 3.2 servings/day; and for ELSA-Brasil, ≥ 566 vs ≤ 235 g/day.

T2DM: type 2 diabetes.
